# Pulpitis

**Published:** 1886-07

**Authors:** 


PULPITIS.

Dr. Geo. Watt objects to the term *pulpitis* to indicate inflammation of the dental pulp. He says that according to the principles of etymology it has no right to exist, and that nothing analogous occurs in our language. Its use shows a want of classical education, and it has no similitude with *Gastritis* or *Stomatitis.*

We dislike to differ from so competent an etymologist, but cannot help thinking him mistaken. The term gastric is derived from the French *Gaster,* the stomach. It is not a classical term, and is somewhat obscure in its application, but it has been universally adopted in medicine as the technical term for the stomach. Hence we get "gastric juice," "gastric nerves," "gastric vessels," and *gastritis,* an inflammation of stomach tissues.

*Stomatatis* is a classical term. It is derived from the Greek, *Stoma,* a mouth, and the term *stomatitis,* referring to an inflammation of the tissues of the mouth, has no reference whatever to the stomach. Hence "stomachitis" *would* indicate a most glaring "want of classical education."

If "Pulpitis," indicating an inflammation of pulp tissues, has no analogue, what is to be said of the following terms, all well estab-lished in medical nomenclature :

*Laryngitis,* inflammation of the Larynx.
*Iritis,* inflammation of the Iris.
*Vaginitis,* inflammation of the Vagina.
*Urethritis,* inflammation of the Urethra.
*Pleuritis,* inflammation of the Pleura.
*Meningitis,* inflammation of the Meninges.
*Cerebritis,* inflammation of the Cerebrum.
*Parotitis,* inflammation of the Parotid.
*Folliculitis,* inflammation of the Follicles.
*Prostatitis,* inflammation of the Prostate.
*Rectitis,* inflammation of the Rectum.
*Colitis,* inflammation of the Colon.
*Osteitis,* inflammation of the Osseous Tissues.
*Periostitis,* inflammation of the Periosteum.
*Phlebitis,* inflammation of the Veins (Greek, *Phlebo*).
*Tonsillitis,*inflammation of the Tonsils.
*Pueumonitis,* inflammation of the Lungs (Greek, *Pneumon*).

*Bronchitis,* inflammation of the Bronchi.
*Glottitis,* inflammation of the Glottis.
*Enteritis,* inflammation of the Intestines (*Enteron*).
*Duodenitis,* inflammation of the Duodenum.
*Peritonitis,* inflammation of the Peritoneum.
*Neuritis,* inflammation of the Nerves (*Neuron*).
*Nephritis,* inflammation of the Kidney (*Nephrus*).
*Pyelitis,* inflammation of the Pelvis of Kidney (*Pyelus*).
*Arthritis,* inflammation of the Joints (*Arthro*).
*Carditis,* inflammation of the Heart (*Cardia*).
*Pericarditis,* inflammation of the Pericardium.
*Endocarditis,* inflammation of the Endocardium.
*Pancreatitis,* inflammation of the Pancreas, and many more that might be instanced, all words legitimately compounded of the names of the tissues with the termination "itis," which signifies an inflammation. The names of the tissues may be derived from the Greek, Latin, French, German or English. In either case the termination and the compounds have been approved and adopted in medical nomenclature, and hence the term "Pulpitis" to designate the inflammation of the tooth-pulp (Latin, *Pulpa,* a soft mass), is classical, scientific, legitimate and proper, and we think should be accepted and adopted as the correct technical term in dentistry.

